# Palladium/Graphene Oxide Nanocomposite for Hydrogen Gas Sensing Applications Based on Tapered Optical Fiber

**DOI:** 10.3390/ma15228167

**Published:** 2022-11-17

**Authors:** Mohammed Majeed Alkhabet, Zaher Mundher Yaseen, Moutaz Mustafa A. Eldirderi, Khaled Mohamed Khedher, Ali H. Jawad, Saad Hayatu Girei, Husam Khalaf Salih, Suriati Paiman, Norhana Arsad, Mohd Adzir Mahdi, Mohd Hanif Yaacob

**Affiliations:** 1 Wireless and Photonics Network Research Centre, Faculty of Engineering, Universiti Putra Malaysia, Serdang 43400, Selangor, Malaysia; 2 Department of Medical Instrumentations Techniques Engineering, Al-Rasheed University College, Baghdad 10053, Iraq; 3 Civil and Environmental Engineering Department, King Fahd University of Petroleum & Minerals, Dhahran 31261, Saudi Arabia; 4 Department of Chemical Engineering, College of Engineering, King Khalid University, Abha 61421, Saudi Arabia; 5 Department of Civil Engineering, College of Engineering, King Khalid University, Abha 61421, Saudi Arabia; 6 Department of Civil Engineering, High Institute of Technological Studies, Mrezgua University Campus, Nabeul 8000, Tunisia; 7 Faculty of Applied Sciences, Universiti Teknologi MARA, Shah Alam 40450, Selangor, Malaysia; 8 Department of Computer Engineering, Federal Polytechnic Mubi, Mubi 650113, Adamawa State, Nigeria; 9 Department of Computer Engineering Techniques, Al-Rasheed University College, Baghdad 10053, Iraq; 10 Department of Physical, Faculty of Science, Universiti Putra Malaysia, Serdang 43400, Selangor, Malaysia; 11 Department of Electrical, Electronic and System Engineering, Faculty of Engineering and Built Environment, Universiti Kebangsaan Malaysia, Bangi 43600, Selangor, Malaysia

**Keywords:** hydrogen gas, tapered optical fiber, graphene oxide, palladium, drop-casting

## Abstract

Gaseous pollutants such as hydrogen gas (H_2_) are emitted in daily human activities. They have been massively studied owing to their high explosivity and widespread usage in many domains. The current research is designed to analyse optical fiber-based H_2_ gas sensors by incorporating palladium/graphene oxide (Pd/GO) nanocomposite coating as sensing layers. The fabricated multimode silica fiber (MMF) sensors were used as a transducing platform. The tapering process is essential to improve the sensitivity to the environment through the interaction of the evanescent field over the area of the tapered surface area. Several characterization methods including FESEM, EDX, AFM, and XRD were adopted to examine the structure properties of the materials and achieve more understandable facts about their functional performance of the optical sensor. Characterisation results demonstrated structures with a higher surface for analyte gas reaction to the optical sensor performance. Results indicated an observed increment in the Pd/GO nanocomposite-based sensor responses subjected to the H_2_ concentrations increased from 0.125% to 2.00%. The achieved sensitivities were 33.22/vol% with a response time of 48 s and recovery time of 7 min. The developed optical fiber sensors achieved excellent selectivity and stability toward H_2_ gas upon exposure to other gases such as ammonia and methane.

## 1. Introduction

The hydrogen (H_2_) content of the atmosphere is in trace amounts (1 ppm by vol.); it is the most prevalent element on Earth, mostly found with oxygen in water and hydrocarbons [1,2,3]. Since hydrogen has a high energy content of 142 kJ/g [4], it can be an essential energy carrier for future renewable energy sources [5,6]. It is viewed as a potential substitute for depleting fossil fuels [7]. It is possible to produce hydrogen fuel through the electrolysis of water, breakdown of hydrocarbon via thermoplastics, or high-temperature steam moving overheated coal [8]. Since hydrogen and oxygen combine to produce energy and water as a by-product, it is considered an environmentally beneficial energy source [9,10]. There are numerous industries where hydrogen is used [11,12,13,14,15,16]; for instance, it is utilized in oil refining, liquid rocket propulsion, and cryogenic research for superconducting studies [17]. It is also used during welding for metal heat treatment, cutting, and coating with atomic hydrogen [18].

Currently, many different technologies are being used or under development for the detection of hydrogen, including semiconductor sensors, electrochemical, thermal sensors, and mass spectroscopy [19,20]. Unfortunately, these sensing methods have several disadvantages, such as large size, high cost, dependence on the presence of oxygen, and the potential to create electrical sparks that would be dangerous in explosive environments. There are four main hydrogen sensor types: chemical resistance, surface acoustic waves, optical fiber sensor, and microelectronic sensor [21].

However, these sensors have significant drawbacks, including low selectivity, high power consumption, and electromagnetic interference (EMI), which restrict their utility in sensing applications. Furthermore, all of the above sensors require higher working temperatures (>300 °C) and a large number of moving mechanical parts, which causes implementation issues in remote sensing applications [22]. To overcome these drawbacks, optical fibers have been investigated as an alternative to conventional chemical sensors due to their numerous benefits. Optical fiber sensors with distributed remote-control features may be readily incorporated into operational networks and communication systems [23]. Chemical-based optical fiber sensors have recently gained a lot of interest because of their compact size, immunity to electromagnetic interference, and ability to operate in harsh environments [24]. Since then, optical fiber chemical sensors have been introduced in various applications, and tremendous efforts have been made to improve their performance. Therefore, the author believes that by using optical fiber sensors in a simpler and low-cost manufacturing process in volatile environments, the safety risks associated with H_2_ leaks can be significantly reduced.

Pallidum (Pd) is a soft, silver–white metal belonging to group 10 of the periodic table. The ability of Pd to absorb H_2_ has been well-studied up to approximately 900 times its volume [25]. Pd has fast H_2_ uptake/sorption kinetics, high H solubility, and selectivity, which makes the catalyst a popular choice for H_2_ optical sensor materials. Several researchers [26,27,28] have developed H_2_ sensors by coating optical fibers with a layer of Pd. In our previous work [29], a Pd-based tapered optical fiber was created and tested at room temperature with various H_2_ gas concentrations in synthetic air. According to the findings, when exposed to 2.00% H_2_ in synthetic air, the Pd-coated based sensor’s absorbance response changed by 63%. The times for response and recovery were 50 and 230 s, respectively. Villatoro et al. [30] further explored this idea to produce a Pd-coated tapered MMF where the waist diameters of tapered sections are between 30 and 70 μm, with the length being 10 mm. The Pd-sensing layer has a thickness of 14 nm. The H_2_ concentration capable of being detected by the sensor was in the range of 0.3–3.5% (*v*/*v*) H_2_ at standard conditions.

Graphene oxide (GO) has recently been in the spotlight as a suitable nanomaterial that can improve sensor performance due to its unique chemical and optical properties, strong water nature, and large surface area [31]. Being a two-dimensional structure, the entire volume of GO is exposed to its environment, which makes it a better candidate for the application of chemical detection [32]. A few research studies have focused on GO as a sensor in layer-based H_2_ sensor applications. Wang et al. [33] fabricated a hydrogen sensor integrated into GO nanostructures using AC DEP technology, a simple and economical manufacturing process. More recently, ZnO MW was synthesized and coated with GO by Rush et al. [34] and showed extremely low power consumption (60–200 nW) and excellent H_2_ detection properties at room temperature

Compared to one-dimensional sensor materials that include nanowires and nanotubes, graphene oxide has clear advantages as a support material for hydrogen detection; it has a perfect two-dimensional structure with a theoretical maximum specified surface area, high load movement, and remarkable structural flexibility [31]. Modifying the surface of graphene is a good way to obtain H_2_-sensitive detection. Several catalytic hydrogen strategies have been developed to enhance noble metal nanoparticles to enhance the response, as the direct interaction of H_2_ with unmodified graphene oxide is too weak to elicit a significant response [35]. The palladium metal catalytic nanoparticles that coated the surface of graphene oxide with a chemical or physical functionalization can improve the detection response due to their high efficiency of dissociation of molecular hydrogen in the most reactive atomic form [36].

Field emission scanning electron microscopy (FESEM), energy dispersive X-rays (EDX), X-ray diffraction (XRD), and atomic force microscopy (AFM) were used in this investigation to characterise the materials. These characterization methods are used to determine the morphology, stoichiometry, composition, crystal structure, and optical features of the produced nanostructured thin films.

To the best of our knowledge, the authors can definitively state that this work is the first to assess the performance of a GO composite integrated with Pd metal nanoparticles coated with tapered optical fiber. Hence, it is an important topic of research that attempts to analyse the tapered optical fiber-based H₂ gas sensor using Pd/GO nanocomposites, followed by the assessment of the properties shown by the nanocomposites as well as the functioning of the newly developed sensor. The performance of the new sensor would be investigated based on different parameters, such as response time, recovery time, repeatability, sensitivity, and stability.

## 2. Materials and Methods

### 2.1. Design and Fabrication of Optical Fiber Transducers

In this research, the author modified the optical fibers with tapering technology. By tapering, the resulting evanescent field was expected to be sufficient for the sensor to perform well. The tapered area will also become sensitive to its circumference, which is good for detection. The tapered optical fibers were coated with a nanostructured material and then showed an optical signal response when exposed to H_2_ in different concentrations.

**Figure 1:**
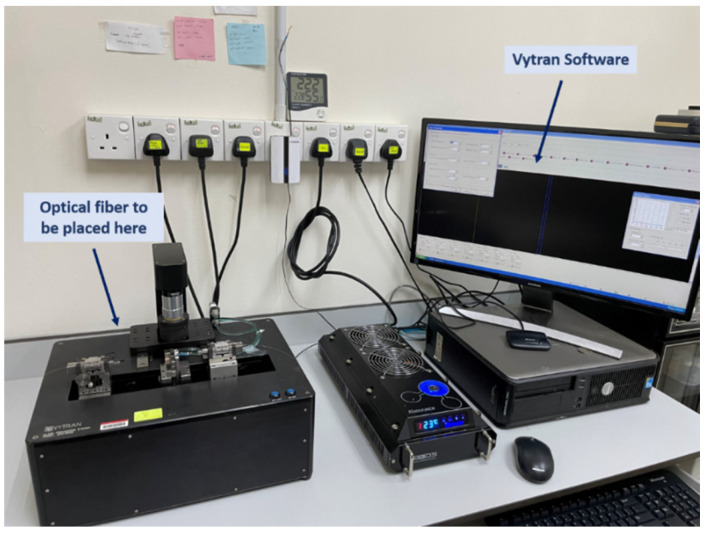
Vytran GPX-3400 glass processing workstation in Photonics Laboratory, Universiti Putra Malaysia (UPM).

The author used a multimode silica (SiO_2_) optical fiber purchased from OFS Furukawa. The diameters of the fiber cladding and core are 62.5 μm and 125 μm, respectively. The wide diameter of the multi-mode optical fiber core enables a strong interaction of light with the sensor layer coated in the tapering region. Furthermore, its small size, light weight, and resistance to EMI are also possible for this fabricator’s sensor application used in the visible wavelength range of the near-infrared. Multimode optical fiber provides low attenuation loss and is flexible at high temperatures, making it attractive for a hydrogen sensor in harsh environments. As a result, the properties of silica optical fiber make it an excellent option for high-sensitivity sensors, remote sensing applications, and high-temperature operation [37]. A multimode fiber using a Vytran GPX-3400 glass processing workstation, as shown in Figure 1, were used for the tapered process.

**Figure 2:**
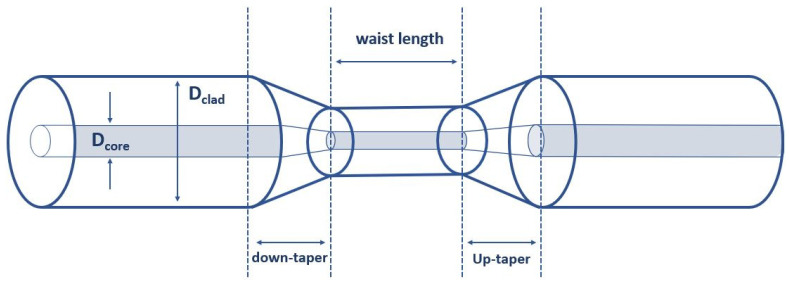
A sketched figure of tapered fiber profile with a uniform waist.

The protective jacket, about 3 cm long, is removed in the tapering step. The tapering procedure is generally performed by pulling and heating the optical fibers. Under a certain pulling force, the optical fiber is stretched and gradually elongated to a desired length and diameter. The shape of the tapered optical fiber can be established by the duration, temperature, and optical fiber tension during the heating step. Argon gas is used in heating to provide an inert local atmosphere. A schematic model of the tapered fiber profile used in this experiment is shown in Figure 2. After the tapering process, the fibers consist of three continuous segments: one tapered waist length segment with a uniform diameter is located in the central region, and is surrounded by two transition regions, called the up-taper and the down-taper regions, whose diameters are gradually changed.

**Figure 3:**
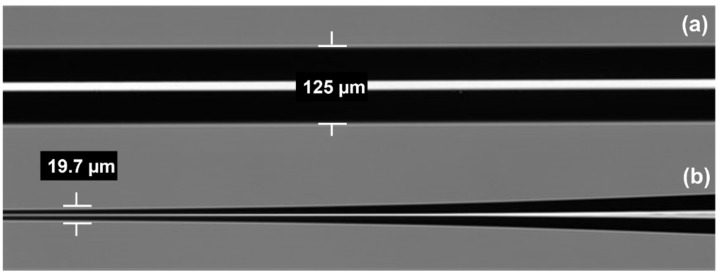
FESEM image of (**a**) Untapered multimodal fibers (MMF) and (**b**) a transition region of a tapered MMF.

The MMF optical fiber was tapered using this machine to produce various modified optical fiber transducing platforms. The author has a varied parameter from waist diameter to 20 μm with a fixed length of 10 mm and an up/down taper of 5 mm, that the size of the waist diameter of the tapered optical fiber will affect the sensitivity of the sensor. This is due to the depth with which the evanescent field penetrates the coated sensor layer [38]. According to [39], the waist diameter range strongly responds to the gas sensor. Figure 3 illustrates the FESEM images of an optical fiber before and after tapering. After the tapering process, the tapered optical fibers were coated with the Pd/GO nanocomposites sensor layer by a drop casting technique.

### 2.2. Synthesis and Deposition of Pd/GO Nanocomposite

In [40], GO was synthesized from modified graphite flakes using the modified hummers process. When the temperature was below 20 °C, the procedure comprised whirling concentrated sulfuric acid with 10 g of graphite powder. Potassium permanganate (KMnO_4_), a strong oxidant, was progressively added to the reaction chamber while it was immersed in an ice bath for two hours. The combination was mixed for 96 h at room temperature. The reaction system was then filled with diluted sulfuric acid (5.0 wt.%) and hydrogen peroxide. After centrifuging and washing the mixture with hydrochloric acid and deionized water to neutralize the filter and remove any remaining contaminants, it was allowed to dry at room temperature. To produce graphite oxide, the precipitate was dried at room temperature. 200 mg of produced graphite oxide powder was dissolved in 800 mL of deionized water. A two-hour ultrasonic treatment was used to exfoliate the graphite oxide dispersion into individual graphene oxide layers.

**Figure 4:**
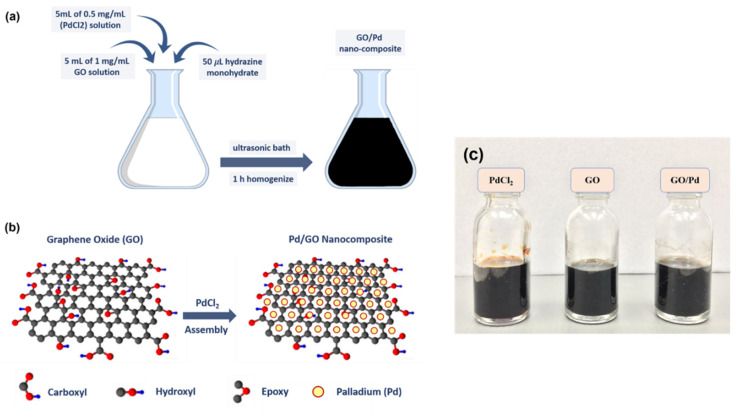
(**a**) Synthesis process of Pd/GO nanocomposite, (**b**) Schematic view of the Pd/GO nanocomposite setting, and (**c**) The stable solution of PdCl_2_, GO, and Pd/GO nanocomposite.

A simple one-step procedure was used to produce Pd/GO nanocomposites. First, a 1 mg/mL palladium chloride (PdCl_2_) solution and a 1 mg/mL GO solution were mixed, followed by 50 µL of hydrazine monohydrate as a reducing agent [41]. After aggressively stirring the resulting solution for 1 h, a stable 10 mL black suspension was obtained. The solution was placed in an ultrasonic bath for an hour to homogenize. The schematic diagram of the Pd/GO nanocomposite is shown in Figure 4.

**Figure 5:**
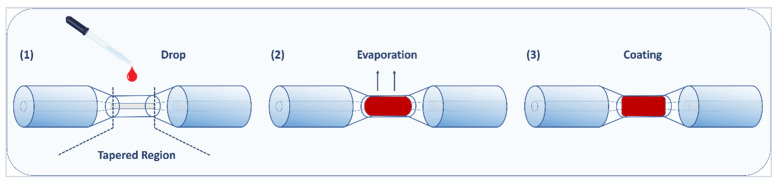
Tapered optical fiber functionalization with Pd/GO nanocomposite using the drop-casting method.

The tapered optical fiber was coated with Pd/GO nanocomposite using a drop-casting technique, in which about 10 µL of the solution was dropped on the fiber base using a tiny pipette and heated to 60 °C in the oven for 20 min to guarantee full evaporation of the aqueous medium [42]. Figure 5 depicts the drop-casting approach used to functionalize a tapered optical fiber with Pd/GO nanocomposites.

### 2.3. Optical Gas Testing Setup

**Figure 6:**
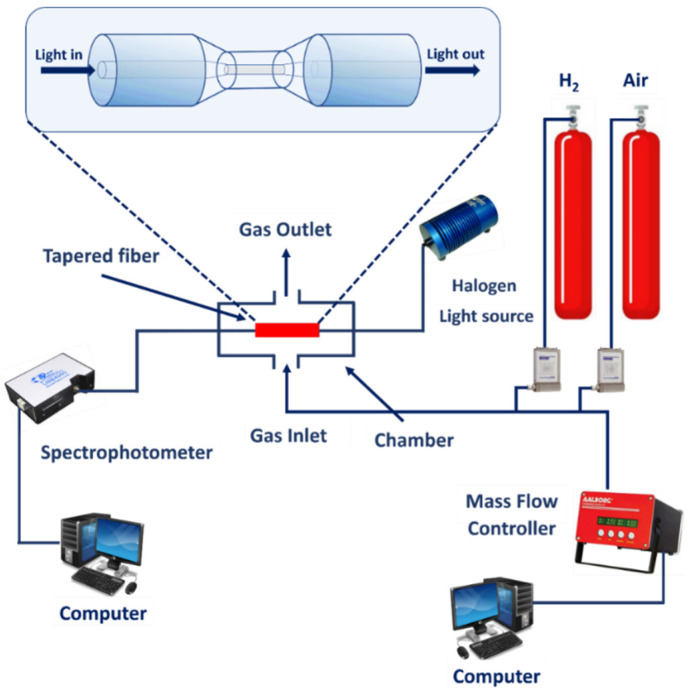
Absorbance measurement gas testing setup.

The absorbance measurement method was employed by the author in this experiment. The investigation concentrated on how the sensing material’s optical absorption spectra responded to H_2_. Figure 6 depicts the setup of the experiment. The proposed H_2_ sensor was installed in a dedicated chamber and optical fiber cables were used to connect it to the tungsten halogen light on one side (Ocean OpticsTM HL 2000, with a wavelength range of 360 to 2400 nm). To measure absorbance, a spectrophotometer (Ocean Optics TM USB-4000, spectral range 200–1100 nm) was connected to the developed sensor. A USB port was used to connect the spectrophotometer to the computer. SpectraSuite (version 6.2) was used to process and evaluate the optical response as it was being measured in real-time with a spectrophotometer. The following formula was used by the software to determine the absorbance Aλ [43]:

(1) $$A_{\lambda }=\;-\log\left(\frac{S_{\lambda }-\;D_{\lambda }}{R_{\lambda }-\;D_{\lambda }}\right)$$

where *S_λ_* represents the intensity of light detected at the wavelength *λ* after being exposed to H_2_, *D* represents the intensity stored when no light goes through the fiber, and *R* represents the reference density of the blank fibers at the wavelength.

A computerized gas calibration system controlled the type and concentration of gas flowing into the chamber (Aalborg Instruments and Controls, Inc., Dunedin, FL, USA). High precision gas concentration control can be achieved with this gas setting. Configuration 1 was connected to the gas titration system using a computer-controlled mass flow controller with a flow rate of 200 m^3^/min. In this graph, the multi-channel gas calibration setup was based on the volumetric mixing of the gases. Certified synthetic air and H_2_ gas cylinders (Linde, Malaysia-Singapore Sdn. Bhd, Petaling Jaya, Selangor, Malaysia) were used to mix and purify the gases in the chamber. The absorbance spectrum was taken from the proposed probes using a spectrophotometer and stored with air as a reference. In the room, diluted H_2_ gas was purified with various concentrations and 100% synthetic air alternatively, while SpectraSuite was analysed and exhibited absorbance with wavelength. The amounts of H_2_ gas were changed from 0.125% to 2.0% to test the sensor’s sensitivity and capacity to measure concentrations. Changes in absorbance have been used to study the sensor’s dynamic responses (changes in absorbance over time).

**Figure 7:**
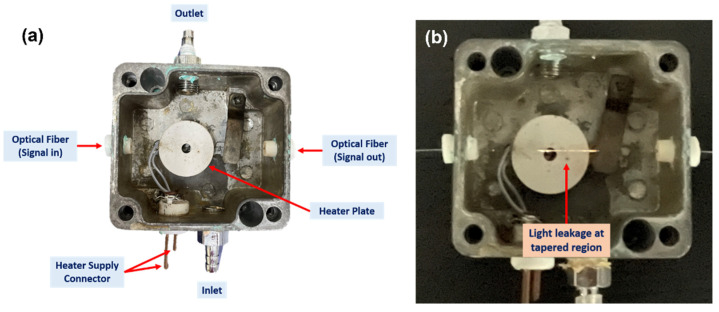
(**a**) Gas chamber for optical fiber and (**b**) light propagates into the tapered optical fiber sensor.

Figure 7a shows the modified gas chamber used for H_2_ sensing. The chamber consists of a heating plate connected to two electrical points connected to the power supply, two ports for gas inlet and outlet, and a thermocouple input. The teflon was drilled on both sides with a hole that meets the optical fiber size. The optical fiber sensor was spliced with a pigtail and connected to the light source and spectrophotometer using an FC-SMA connection. Figure 7b depicts the light that propagates into the optical fiber’s tapered area. According to the image, when exposed to H_2_, the leaking light interacts with the sensor layer and modifies its characteristics.

## 3. Characterisation and Chemical Sensing Results

### 3.1. Micro-Characterizations of Pd/GO Nanocomposite

**Figure 8:**
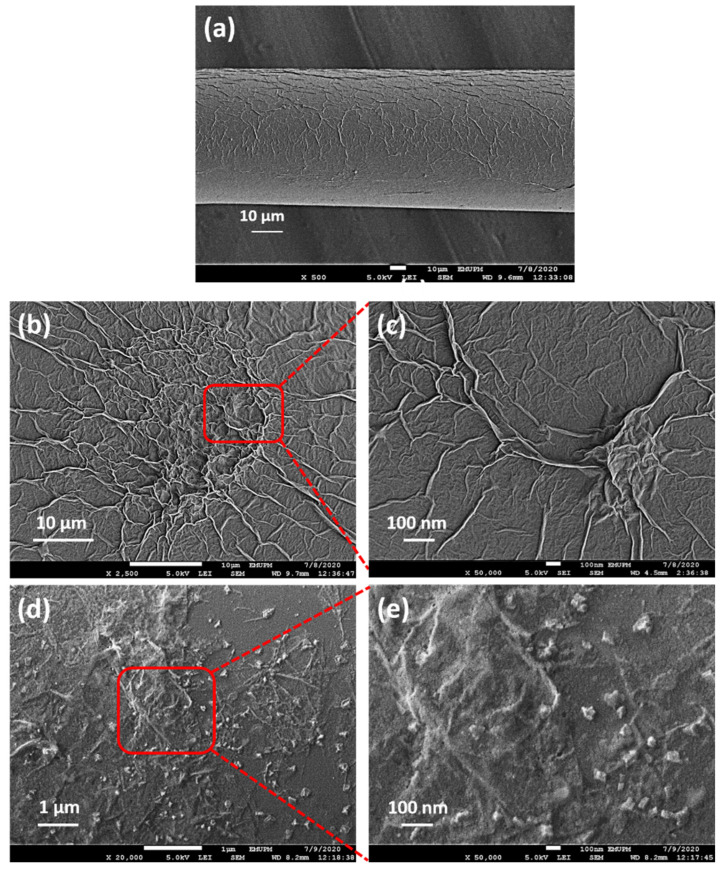
FESEM micrographs of (**a**) tapered MMF coated with Pd/GO composite, (**b**,**c**) GO, (**d**,**e**) Pd/GO nanocomposite.

The author performed the FESEM characterisation of the samples to analyse the GO and Pd/GO nanocomposite morphological characteristics. Figure 8a depicts the successful deposition of the GO and Pd/GO nanocomposite on the surface of the tapered fiber. The coated GO surface was relatively smooth, with a wrinkled structure that might be related to the edges of the GO sheets [44]. As demonstrated in Figure 8, wrinkles are significant for GO surfaces because they provide a large area of surface for robust sensing capability (Figure 8b,c). Furthermore, the picture clearly shows that the GO distribution is uniform, as shown in [45,46].

Regarding Pd/GO nanocomposite films, the Pd NPs on the surface of GO can be identified in Figure 8d,e. It shows a uniform distribution of Pd particles on the GO sheets and indicates that Pd mixes and diffuses uniformly through GO. According to [47,48], the wide aggregation can be attributed to the unequal size of the Pd NPs and the folding of the GO nanoparticles since the small particles interfered with the larger particles in the nanocomposite solution of Pd/GO. The dispersion quality of the Pd particles in the GO matrix significantly influences hydrogen storage in the nanocomposite [49].

**Figure 9:**
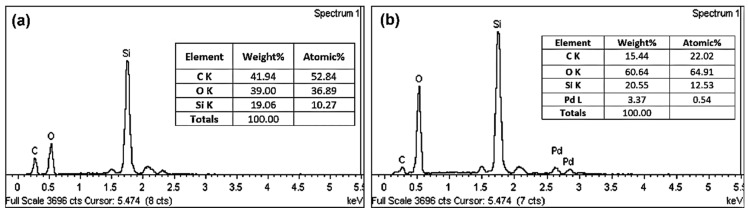
EDX measurement of (**a**) GO and (**b**) Pd/GO nanocomposite.

The elemental composition of the GO and Pd/GO nanocomposite were measured by EDX performed in FESEM. The EDX results confirm that the present elements of GO are C, O, and Si, as shown in Figure 9a. At the same time, the EDX result of the Pd/GO nanocomposite was C, O, Pd, and Si in the synthesized nanocomposite, as shown in Figure 9b. The silica fibers used the silicon peak (Si) as a substrate.

**Figure 10:**
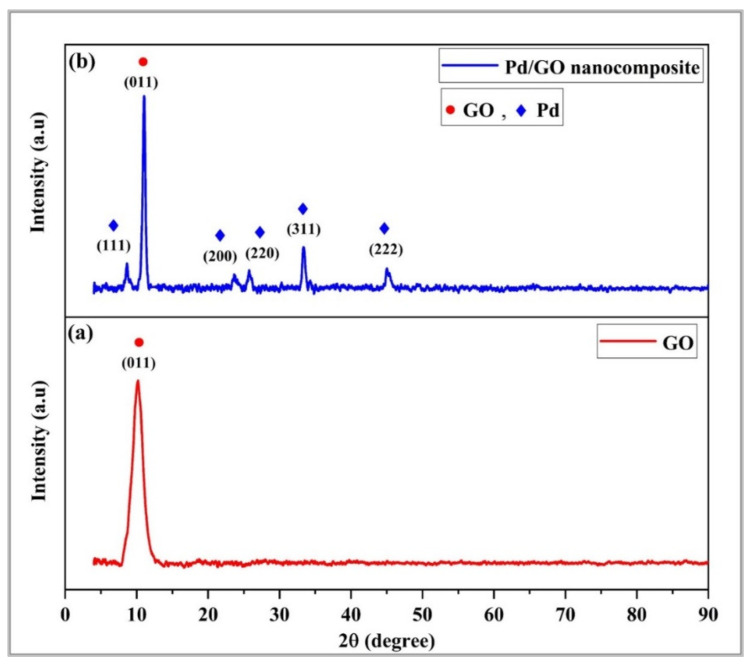
XRD pattern for both samples (**a**) GO thin film and (**b**) Pd/GO nanocomposite thin film.

According to Figure 10, the GO molecule exhibits a distinct intensity peak of 2θ at a value of 10.11, which corresponds to the (011) plane. The larger spacing of GO [50] may be the cause of the existence of oxygen-containing groups like hydroxyl, carboxylate, and epoxy. The spacing between the GO layers was 0.865 nm according to using Bragg’s law [51]:

λ = 2dsin θ(2)

where λ is the X-ray beam’s wavelength (0.154 nm). The d-range of graphene oxide interlayers ranges from 0.6–1.0 and is controlled by the degree of oxidation of graphite and the number of water molecules intercalated in the interlayer apace [52].

The peaks stated in Figure 10, as well as the strong Pd peaks, are maintained similarly in the GO and Pd/GO nanocomposite samples. This suggests that the Pd nanoparticles were successfully fixated to the Pd/GO matrix. The Pd/GO nanocomposite diffraction pattern revealed a crystal peak value of 2 of 8.63°, 25.75°, 28.55°, 33.40°, 45.01° and matching to the (111), (200), (220), (311) and (222) surfaces of the Pd lens’s central cubic structure, respectively. This is consistent with the known literature [53]. The greatest signal was seen at 2 = 33.40°, indicating that Pd nanoparticles had been incorporated into the GO sheets in the dominant plane (311). As can be observed from the XRD profile of the Pd/GO nanocomposite, the peaks gradually became shorter.

The nanocomposite’s GO pattern has an intense peak at 11.80°, which corresponds to an interlayer spacing of 0.55 nm. Because of the addition of oxygenated groups and ultrasonically assisted exfoliation, this is narrower than GO [54].

**Figure 11:**
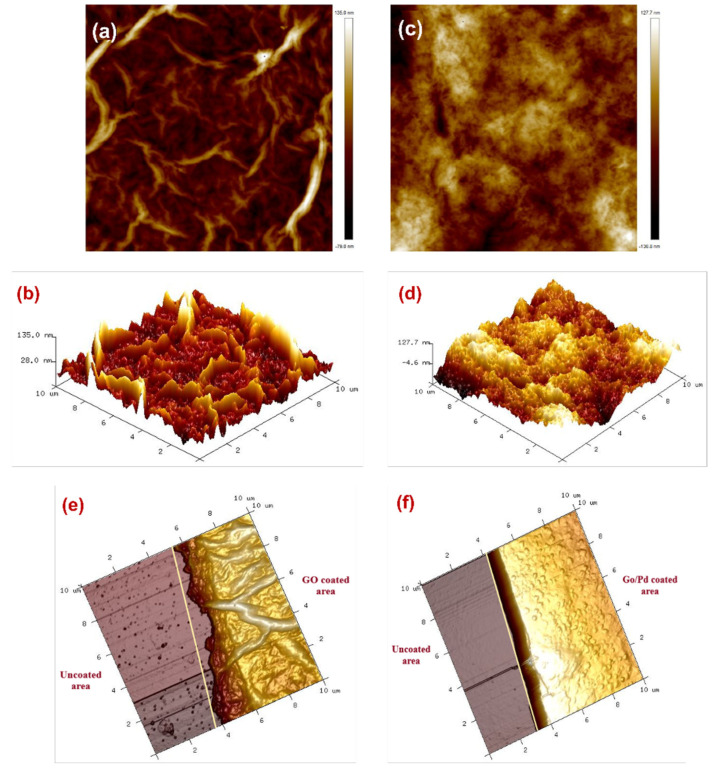
2D topography of AFM images of (**a**) GO and (**c**) Pd/GO composite. 3D AFM images of (**b**) GO and (**d**) Pd/GO composite. 3D AFM topography images of the boundary region between the uncoated and coated fibers of (**e**) GO and (**f**) the Pd/GO composite sensor layer.

The surface roughness and thickness of GO and Pd/GO compounds are verified using atomic force microscopy (AFM). The border region was allocated a 10 m × 10 m scan for AFM analysis. The average surface roughness of the GO and Pd/GO compounds was 26 and 30 nm, respectively, as shown in Figure 11. The coarse sensing layer is important because it allows gas molecules to easily diffuse into and out of the layer. As a result, sensing element performance parameters such as reaction time and sensitivity can be significantly improved. The thickness of the thin films of the GO and Pd/GO nanocomposite were measured. A portion of the substrate was covered with aluminium tape to differentiate between coated and uncoated sites during the coating process. The thickness of the GO and Pd/GO composite coatings were measured as 610 nm and 850 nm, respectively.

### 3.2. Hydrogen (H_2_) Response-Based Pd/GO Nanocomposite

Before performing any H_2_ testing on the fabricated optical sensor, the original substrate’s preliminary characteristic must be identified first. The uncoated or blank untapered and tapered optical multimode fiber were tested towards H_2_ to check whether any response was detected from the substrate. The diameter of untapered and tapered blank optical fiber was 125 µm and 20 µm, respectively.

**Figure 12:**
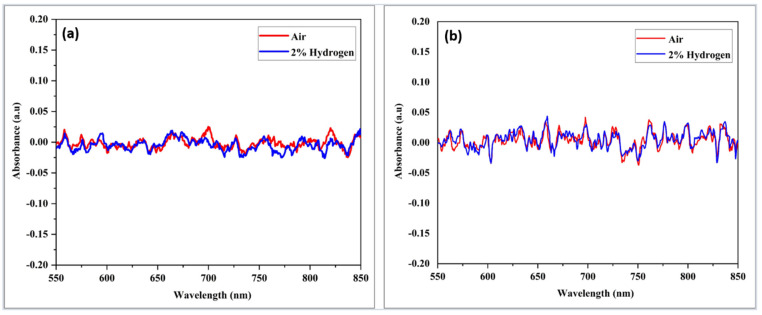
Absorbance response versus wavelength for multimode optical fibers (**a**) untampered and (**b**) tapered when exposed to 2.00% H_2_ at room temperature.

**Figure 13:**
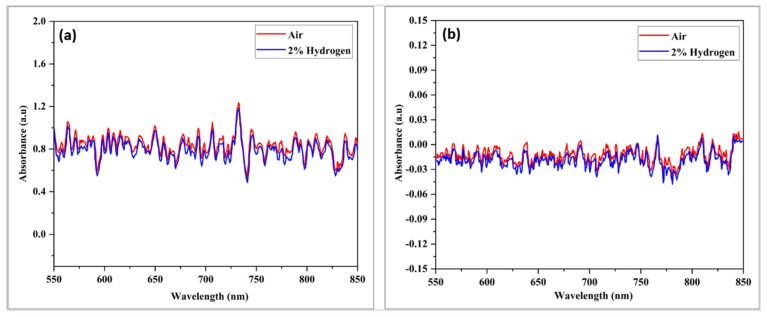
Absorption response versus uncoated wavelength (**a**) untapered, (**b**) tapered multimodal optical fibers when exposed to 2.00% H_2_ at 200 °C.

The blank samples were exposed to 2.00% H_2_ at room temperature, as depicted in Figure 12. It was observed that there was no change in absorbance magnitude for both samples when 2.00% of H_2_ was purged in the chamber. No response was recorded when the test was continued at the high operating temperature of 200 °C, as demonstrated in Figure 13. It can be concluded that tapered and non-tapered optical fibers do not respond to, H_2_ since there was no interaction between the optical fiber and H_2_. Coating tapered optical fibers with nanostructured materials is expected to improve the sensitivity of its absorbance change toward the H_2_ environment.

**Figure 14:**
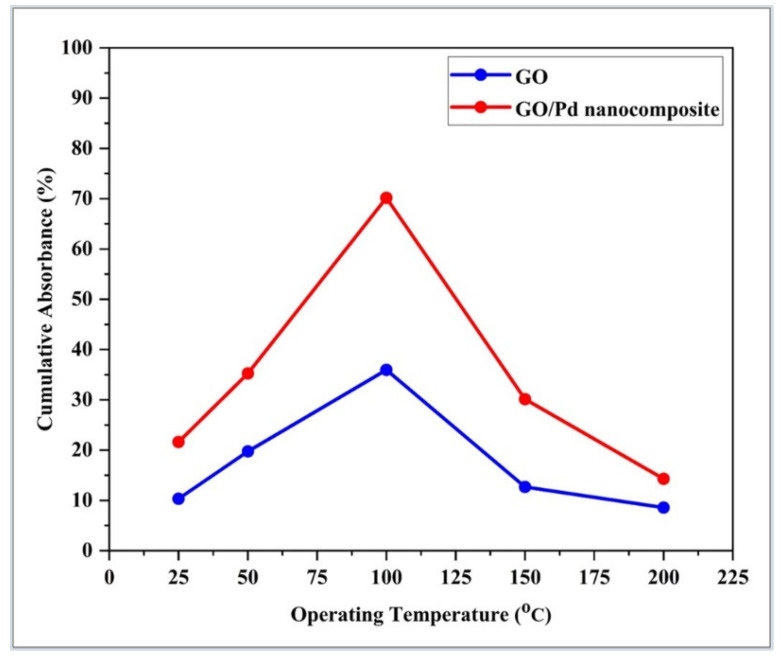
The change of cumulative absorption with an operating temperature of GO and Pd/GO nanocomposite-based optical fiber sensor towards synthetic air and 2.00% H_2_.

The sensor sample was a Pd/GO nanocomposite coated on tapered optical fiber. Figure 14 depicts the change in cumulative absorbance versus operating temperature of GO and Pd/GO nanocomposites coated on tapered optical fibers when exposed to 2.00% H_2_ in synthetic air. A spectrophotometer incorporated a response curve from the 550–850 nm wavelength range to calculate cumulative absorbance. The figure depicts the optimum operating temperature of the GO and Pd/GO nanocomposite at 100 °C. The Pd/GO nanocomposite-based sensor exhibits a higher absorbance change than the GO sample. At lower operating temperatures, low absorbance may be due to slow chemical activation between the sensor layer and the absorbed gas molecule [55]. It can also be shown that the adsorption reaction slows down as temperature increases, possibly due to the increased reaction rate of adsorbed hydrogen atoms on active sites at higher temperatures [56]. The optimal operating temperature for GO and Pd/GO H_2_-based tapered optical fiber sensors for H_2_ was determined as 100 °C.

**Figure 15:**
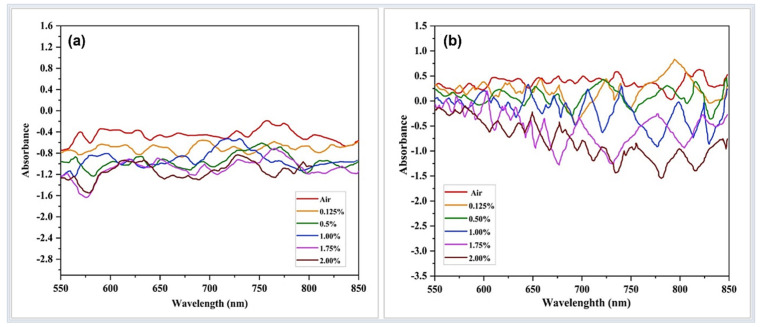
Absorbance versus optical wavelength (**a**) Pd/GO and (**b**) GO-coated optical fiber-based sensor towards synthetic air and 2.00% H_2_ at 100 °C.

Figure 15a,b illustrates the absorbance spectra of the developed tapered optical fiber sensor toward different H_2_ concentrations. A distinctive absorbance change can be observed over the wavelength range of 550 nm to 850 nm. This wavelength is selected as the absorbance spectrum demonstrates the largest response toward H_2_ within this range based on experiments. The absorbance magnitude proportionally increased when H_2_ concentration increased. Between 550 and 850 nm, absorbance changes were associated with the Pd/GO nanocomposite coated sensors relative to the GO coated sensors. Nevertheless, the absorbance changes were higher in the Pd/GO nanocomposite-based sensors, which may be attributable to the presence of OH in GO [57].

**Figure 16:**
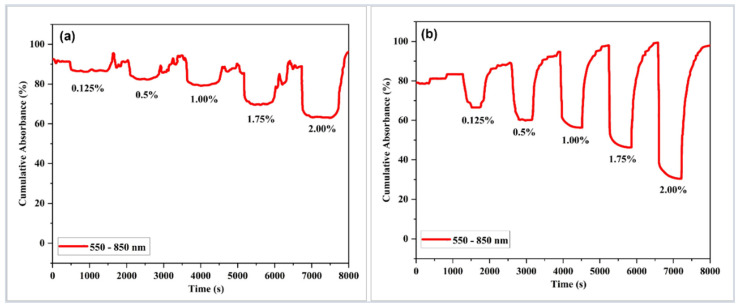
Dynamic absorbance curves of sensors coated with (**a**) GO and (**b**) Pd/GO nanocomposite to different H_2_ concentrations in synthetic air at 100 °C.

The dynamic absorbance responses of a tapered optical fiber coated with GO and Pd/GO nanocomposite thin film to different concentrations of H_2_ gas at 100 °C over the wavelength range of 550–850 nm are shown in Figure 16a,b. As shown in Figure 16a, the dynamic response curve demonstrated that the sensor changed in response to the changing H_2_ concentrations. As a result, the GO-based sensor was not suitable as an active sensing layer for optical gas sensing applications. At a gas level of 0.125% H_2_, the gas absorption in the GO layer showed a change in absorbance of 5%. At a concentration 2.00% higher, the absorbance increased to 35%. The observed response and recovery time for 2.00% H_2_ were calculated as 2 min and 11 min, respectively.

In contrast, the Pd/GO nanocomposite-based sensor demonstrated a superior absorption response with the response and recovery durations of 48 s and 7 min, respectively, compared to the GO-based sensor. Changes in absorption were approximately 14% higher at 0.125% H_2_ concentration and 70% higher at 2.00% H_2_ concentration. As demonstrated in Figure 16, the dynamic response of the Pd/GO nanocomposite-based sensor identified less H_2_ at higher absorption changes than the GO-based sensor Figure 16b. The constructed sensor responds quicker than the optical fiber sensors described by Phan et al. [58]. When synthetic air was pumped into the chamber at 100 °C, the Pd/GO coated-based sensor recovered effectively.

**Figure 17:**
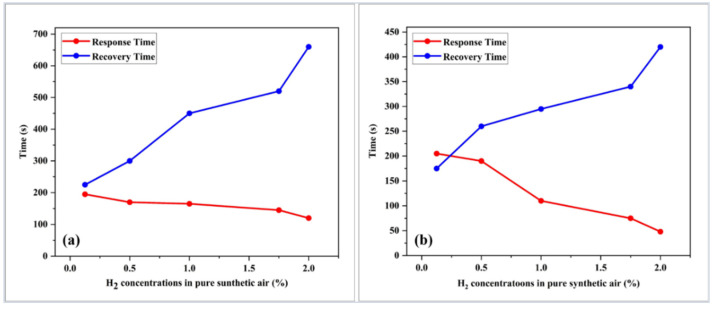
Effect of H_2_ concentrations on the response and recovery times of (**a**) GO and (**b**) Pd/GO nanocomposite-based sensors at 100 °C.

At 100 °C, increasing the H_2_ concentrations resulted in a faster response with a lower recovery time of GO and Pd/GO nanocomposite coated optical fiber-based sensors, as shown in Figure 17a,b. The response time for the Pd/GO nanocomposite-based sensor was decreased from 205 s to 48 s, whereas the recovery time increased from 175 s to 420 s. The response time of the GO-based sensor, decreased from 195 s to 120 s, but the recovery time increased from 225 s to 660 s.

**Figure 18:**
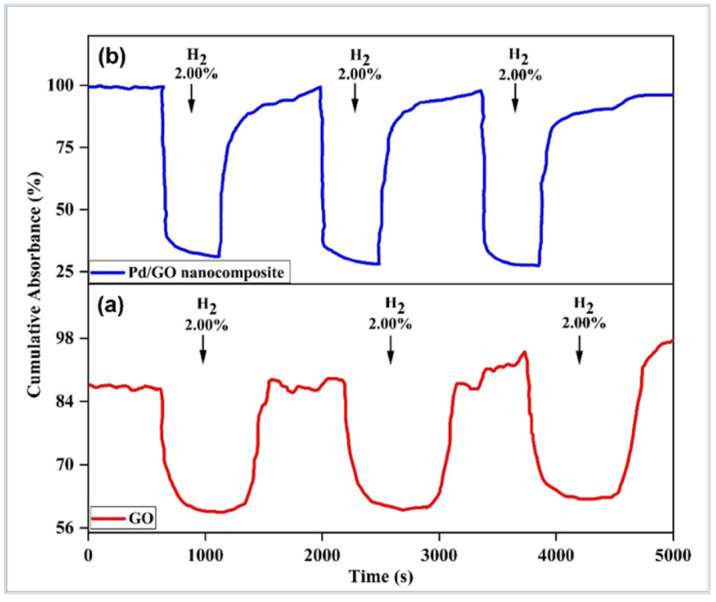
The repeatability of the developed sensor towards 2.00% concentration of H_2_ in synthetic air at 100 °C (**a**) GO and (**b**) Pd/GO nanocomposite-based sensor.

As illustrated in Figure 18, the suggested sensors were tested three times with H_2_ at a 2.00% concentration to determine their repeatability. The sensors’ sensitivity, responsiveness, and recovery times were found to be comparable. Excellent sensor repeatability is demonstrated here, which is crucial for precise H_2_ detection. The response drift that occurred in the GO-based sensor was overcome by tapered optical fibers coated with Pd/GO NC. Overall, the dynamic response of the constructed GO and Pd/GO NC-based sensors demonstrated excellent repeatability at 2.00% H_2_ and a high degree of absorbance.

**Figure 19:**
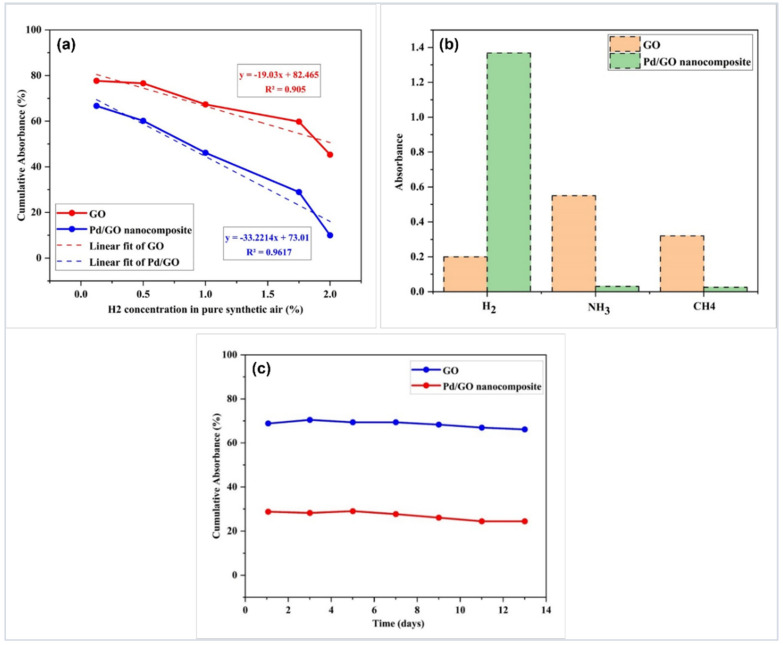
(**a**) Absorbance changes at different H_2_ concentrations for coated GO and Pd/GO nanocomposite-based sensors, (**b**) comparison bar graph of the selectivity of optical fibers coated with GO and Pd/GO nanocomposite-based sensors, and (**c**) stability of the fabricated sensors towards 2.00% H_2_ gas concentration in synthetic air at 100 °C.

Figure 19a depicts the cumulative absorbance change of the GO and Pd/GO nanocomposite sensing layers as a function of H_2_ concentration in the 550–850 nm wavelength range. The decrease in absorbance is proportional to the increase in H_2_ concentration, depending on the figure. The tapered optical fiber sensor-based GO and Pd/GO nanocomposite sensitivities were 19.03/vol% and 33.22/vol%, respectively, with an excellent linear slope of about 90% and 96.3%, respectively. Overall, the Pd/GO nanocomposite-based sensor performed noticeably better than the GO-based sensor.

A selectivity test was also performed for sensors coated with GO and Pd/GO nanocomposites towards ammonia (NH_3_) and methane (CH_4_). The selectivity measurement and comparison of H_2_, NH_3_, and CH_4_ at the same operating temperature of 100 °C at a concentration of 2.00% were shown in Figure 19b. The sensors were evaluated for selectivity in terms of absorbance response for a specific gas under similar conditions. The H_2_ absorbance of the Pd/GO nanocomposite sensor was astonishingly high, but its response to NH_3_ and CH_4_ gases was noticeably weak. Contrarily, the GO sensor responds to NH_3_ and CH_4_ with a greater H_2_ absorbance response. A high working temperature is required to increase the sensitivity of Pd/GO sensor-based H_2_ gas because methane is a stable gas that requires significant energy to completely separate H_2_ from C [59]. The Pd/GO sensor showed less sensitivity to NH_3_ because Pb is better suited for H_2_ gas separation [60]. This confirms that the produced Pd/GO nanocomposite-based sensor demonstrates good H_2_ selectivity compared to the GO-based sensor.

The stability of the GO and Pd/GO nanocomposite-based sensors for 14 days was also measured towards an H_2_ gas concentration of 2.00% in synthetic air at 100 °C, as shown in Figure 19c. The sensors were stored in a closed container at 20 °C in a dry cabinet to avoid contamination of the sensor surface. After two weeks, the gas response was slightly reduced by 5% and 3% for GO, and Pd/GO nanocomposites-based sensors, respectively. This result indicates that the prepared gas sensors, especially the Pd/GO nanocomposite-based sensor, present excellent stability. The test highlights that contribute greatly to the understanding of the properties of the chemical adsorbent towards H_2_ are illustrated in Table 1.

**Figure 20:**
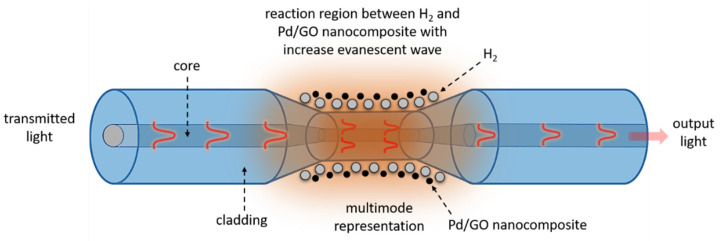
Illustration of gas-sensing mechanism between the H_2_ molecules and Pd/GO nanocomposite on tapered optical fiber.

The sensing mechanism of the Pd/GO nanocomposite-based sensor can be explained in Figure 20. The Pd/GO nanocomposite-based H_2_ sensor mechanism is well-known [41,65,66]. When Pd adsorbs the H_2_ gas molecules, it changes to Pd hydride (PdHx) (where the Pd particle magnitude expands in small proportion), as it has fewer functions than pure Pd. The PdHx feature is handy in transferring more electronics from Pd to GO. In the Pd/GO nanocomposite, it is hypothesized that graphene oxide played a significant role as a support in carrying and detaching Pd nanoparticles on the tapered optical fiber, hence providing channels for the transport of charges. H_2_ molecules during Pd nanoparticles adsorption and H_2_ molecules desorption can diffuse even if Pd nanoparticles are coiled and loaded by graphene oxide due to the atomic thickness of graphene oxide.

## 4. Conclusions

The aim of this study is to develop tapered optical fiber sensors coated with a Pd/GO nanocomposite for H_2_ sensing applications. The H_2_ sensing performance analysis is performed by the investigation of the nanocomposite-coated optical fiber. Characterization technologies include FESEM, EDX, XRD, and AFM. The characterization results confirmed the purity of the nanoparticles and their effective deposition onto the tapered optical fiber. The analysis showed that the performance of the Pd/GO nanocomposite-based sensor was repeatable at gas concentrations of 0.125–2.00% in air at 100 °C. When exposed to various gases such as NH_3_ and CH_4_, the developed optical fiber sensors demonstrated high response, excellent selectivity, and stability toward H_2_ gas.

## Figures and Tables

**Table 1:** Summary and comparison of the developed tapered optical fiber sensors’ sensing performance.

Sensor-Based	Sensor Structure	Operating Temperature °C	Sensitivity (Abs/%)	Response Time (s)	Ref.
Pd NPs	Quartz substrate	RT	15.40	50	[29]
GO	PMMA fiber	RT	2.33	90	[33]
Pd/MnO_2_	Tapered MMF		3.609	240	[61]
Pd NPs	Mach-Zehnder interferometer	RT	2.58	30	[62]
Pd NPs	Tapered MMF	RT	4.285	100	[30]
Pd/Ag	Etched FBG	RT	2.112	280	[63]
Pd/α-MoO_3_	Substrate with Pd electrode	200	1.46	230	[64]
GO	Tapered MMF	RT	19.03	120	This work
Pd/GO nanocomposite	Tapered MMF	RT	33.221	48	This work

Ref. refers to reference and RT refers to reference room temperature.

## Data Availability

Not applicable.
